# The Lens

**Published:** 1872-06

**Authors:** 


THE LENS is the name of a new quarterly Journal of microscopy and the allied natural sciences, published by the state microscopical society of Illinois. Numbers one and two have already been published and present a fine appearance, containing many very fine articles on topics pertaining to microscopy. The Journal is ably edited by S. A. Briggs, office 177 Calumet Avenue, Chicago.

